# Editorial: The neurobiological and cognitive underpinnings of appetitive and aversive motivation

**DOI:** 10.3389/fnbeh.2024.1383393

**Published:** 2024-02-21

**Authors:** Francesca Starita, Yoann Stussi, Sara Garofalo, Damiano Terenzi

**Affiliations:** ^1^Center for Studies and Research in Cognitive Neuroscience, Department of Psychology, University of Bologna, Bologna, Italy; ^2^Swiss Center for Affective Sciences and Department of Psychology, University of Geneva, Geneva, Switzerland; ^3^Institut de Neurosciences de la Timone, UMR 7289 CNRS & Aix-Marseille Université, Marseille, France

**Keywords:** appetitive motivation, aversive motivation, reward, learning, decision-making, cognitive control, ventromedial prefrontal cortex (vmPFC), behavioral neuroscience

Motivation is a complex construct encompassing the processes involved in the initiation, direction, energization, intensity, and persistence of behavior. These processes are central to survival and wellbeing: They help organisms anticipate, adapt, and react to changes in their internal states and in their environment by eliciting multifarious affective responses and appetitive or defensive behaviors (Berridge, 2018; Levy and Schiller, 2021; Stussi and Pool, 2022). For instance, organisms are typically motivated by the opportunity of obtaining rewards and avoiding punishments or threats, which in turn facilitates the execution of approach and avoidance behaviors, respectively (Guitart-Masip et al., 2014; Starita et al., 2022). Whereas, these forms of appetitive and aversive motivation are generally highly adaptive and contribute to healthy functioning, they can sometimes go awry and become maladaptive. This is notably the case in conditions such as apathy, anhedonia, addiction, impulse disorders, or anxiety-related disorders (Berridge and Robinson, 2016; Mogg and Bradley, 2018; Terenzi et al., 2022; Bresin and Hunt, 2023).

In the past decade, a flourishing of studies have examined the behavioral and neural correlates of motivation in healthy individuals as well as the cognitive determinants of motivation and its dysregulation in several psychopathological disorders. With the emergence of affective science (Dukes et al., 2021), there also has been an increased focus and recognition of the prominent influence that motivation exerts on cognition, including attention, learning, memory, and decision-making (Pool et al., 2016; O'Doherty et al., 2017; Stussi et al., 2018; Starita et al., 2019; Garofalo et al., 2020; Terenzi et al., 2021). These two aspects have contributed to pushing the boundaries of knowledge on motivated behavior and the bidirectional links between motivational and cognitive processes.

In this perspective, this Research Topic aims to promote the cognitive neuroscience of human motivation in a 2-fold manner. First, it highlights recent progress in the study of the neural substrates of appetitive and aversive motivation in humans. Second, it showcases how appetitive and aversive motivation impact cognition across various domains. Through these objectives, the present Research Topic seeks to advance the understanding of the neurobiological and psychological mechanisms underlying appetitive and aversive motivation and their influence on cognition.

A key focus of investigation in this Research Topic concerns the role of specific brain regions in reward processing—a core component of appetitive motivation. The brain's reward system involves key structures such as the striatum, the midbrain dopamine neurons, the amygdala, and the prefrontal cortex, including the ventromedial prefrontal cortex (vmPFC; Haber and Knutson, 2010; Berridge and Kringelbach, 2015; Hiser and Koenigs, 2018). While prior research has demonstrated that the vmPFC is involved in the reward network, its precise contribution remains unclear. The study of Rehbein et al. tested the causal role of the vmPFC in predicting and processing rewards by modulating vmPFC excitability with transcranial direct current stimulation (tDCS) before participants performed a gambling task. Results showed that vmPFC excitation through tDCS introduces a positive bias in the reward system, enhancing anticipation, positive outcome appraisal, and improving reward-based learning. This was evident in greater behavioral flexibility following losses and unexpected outcomes, indicating an improved response to feedback. These findings provide evidence delineating the crucial role of the vmPFC in predicting and processing rewards.

Another set of contributions explored the effects of appetitive and aversive motivation on a range of cognitive processes. Gorrino et al. investigated the role of the insular cortex—a key brain region implicated in appetitive and aversive motivated behavior (Ibrahim et al., 2019; Centanni et al., 2021; Horing and Büchel, 2022)—in decision-making and cognitive control. Using high-definition tDCS, they observed no effect of stimulation of the right posterior insula either on loss and risk aversion or on cognitive control. These negative findings highlight the need for further research to establish the effectiveness and sensitivity of insula stimulation in modulating the interplay between motivational and cognitive processes. Yang et al. further examined the influence of aversive motivation on cognitive control. Specifically, they compared the contribution of aversive motivation vs. negative affect to punishment-driven improvement in cognitive control. Using the Stroop Task and manipulating feedback type (punishment vs. neutral) and feedback contingency (performance-contingent vs. non-contingent), they found that negative affect *per se* did not enhance cognitive control. Instead, aversive motivation improved cognitive control, especially for participants who were aware of the contingency between punishment and performance. These results outline the contribution of aversive motivation to cognitive control and uncover the critical role of metacognition in this process. Finally, Bublatzky et al. investigated whether aversive apprehensions, i.e., trait anxiety, affect spatial navigation in threatening or safe virtual reality contexts. They showed that, relative to individuals with lower trait anxiety, more anxious individuals displayed improved spatial navigation only in the threatening context. This threat-related enhancement in spatial navigation was selectively observed in a route retracing task, but not in a route repetition one. These results suggest that, besides their detrimental effects, aversive apprehensions may support navigational performance when congruent with adaptive avoidance behaviors.

In sum, the present Research Topic offers an overview of the current research on appetitive and aversive motivation that features the diversity of theoretical and methodological approaches within this field. We would like to thank the authors for their work, which provides new insights into the functioning of human motivation. Further exploration into the dynamics of appetitive and aversive motivation may pave the way for informing the development of targeted interventions, thereby fostering a deeper integration of basic and clinical research. This could ultimately contribute to better identifying the role of maladaptive motivational processes in the etiology and maintenance of mental health issues and how these processes can be targeted to improve wellbeing. We hope that this Research Topic will spark interest toward this goal and toward the continuous blossoming of cutting-edge research on human motivation.

## Author contributions

FS: Writing—original draft, Writing—review & editing. YS: Writing—original draft, Writing—review & editing. SG: Writing—review & editing. DT: Writing—original draft, Writing—review & editing.
